# Electronically Perturbed Vibrational Excitations of the Luminescing Stable Blatter Radical

**DOI:** 10.1021/acsnano.4c09661

**Published:** 2025-02-21

**Authors:** Jonathan Bar-David, Abdalghani Daaoub, Shangzhi Chen, Sarah May Sibug-Torres, Sara Rocchetti, Gyeongwon Kang, Ross J. Davidson, Rebecca J. Salthouse, Chenyang Guo, Niclas Sven Mueller, Sara Sangtarash, Martin R. Bryce, Hatef Sadeghi, Jeremy J. Baumberg

**Affiliations:** † NanoPhotonics Centre, Cavendish Laboratory, Dept. of Physics, University of Cambridge, Cambridge CB3 0HE, U.K.; ‡ Device Modelling Group, School of Engineering, University of Warwick, Coventry CV4 7AL, U.K.; § Dept. of Chemistry, Durham University, Durham DH1 3LE, U.K.

**Keywords:** nanophotonics, SERS, radicals, SAM, electrochemistry, nanoparticles, Raman spectroscopy

## Abstract

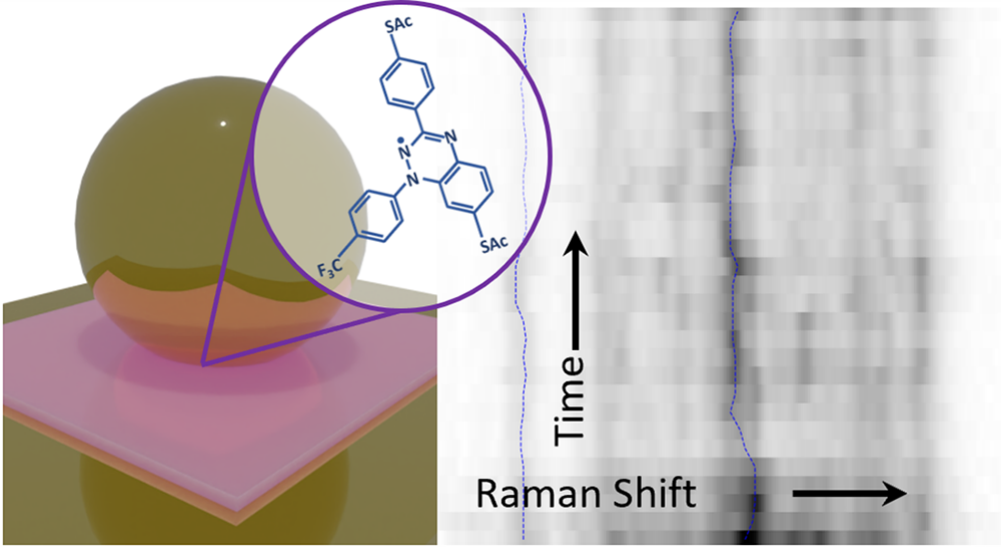

Stable radicals are spin-active species with a plethora of proposed applications in fields from energy storage and molecular electronics to quantum communications. However, their optical properties and vibrational modes are so far not well understood. Furthermore, it is not yet clear how these are affected by the radical oxidation state, which is key to understanding their electronic transport. Here, we identify the properties of 1,2,4-benzotriazin-4-yl, a stable doubly thiolated variant of the Blatter radical, using surface-enhanced Raman scattering (SERS). Embedding molecular monolayers in plasmonic nanocavities gives access to their vibrational modes, photoluminescence, and optical response during redox processes. We reveal the influence of the adjacent metallic surfaces and identify fluctuating SERS signals that suggest a coupling between the unpaired radical electron and a spatially overlapping vibrational mode. This can potentially be exploited for information-storage devices and chemically designed molecular qubits.

## Introduction

Radical molecules possess at least one unpaired electron,^1−3^ but while most exist only for short times as intermediates during chemical reactions, some radicals are stable in ambient conditions, both in the crystalline phase, when dissolved in solution,^1−3^ or assembled on surfaces.^4^ The strategies that stabilize radical species include steric hindrance, electron delocalization, atom electronegativity, and multiple heteroatoms, all preventing the lone electron from reacting to form new bonds.^1−3,5^ Due to the unpaired electron, radical molecules possess magnetic moments, making them interesting as local probes and for various applications such as magnetic markers for electron paramagnetic resonance (EPR) in life sciences,^1−3,6,7^ as energy-storage materials for batteries,^1−3,8^ for thermo-voltaic materials,^1−3,9−11^ molecular electronics,^12−14^ and for quantum computation.^15−20^ Organic radicals are of particular interest due to their potential for replacing rare-earth elements used in current technologies.

**Figure 1:**
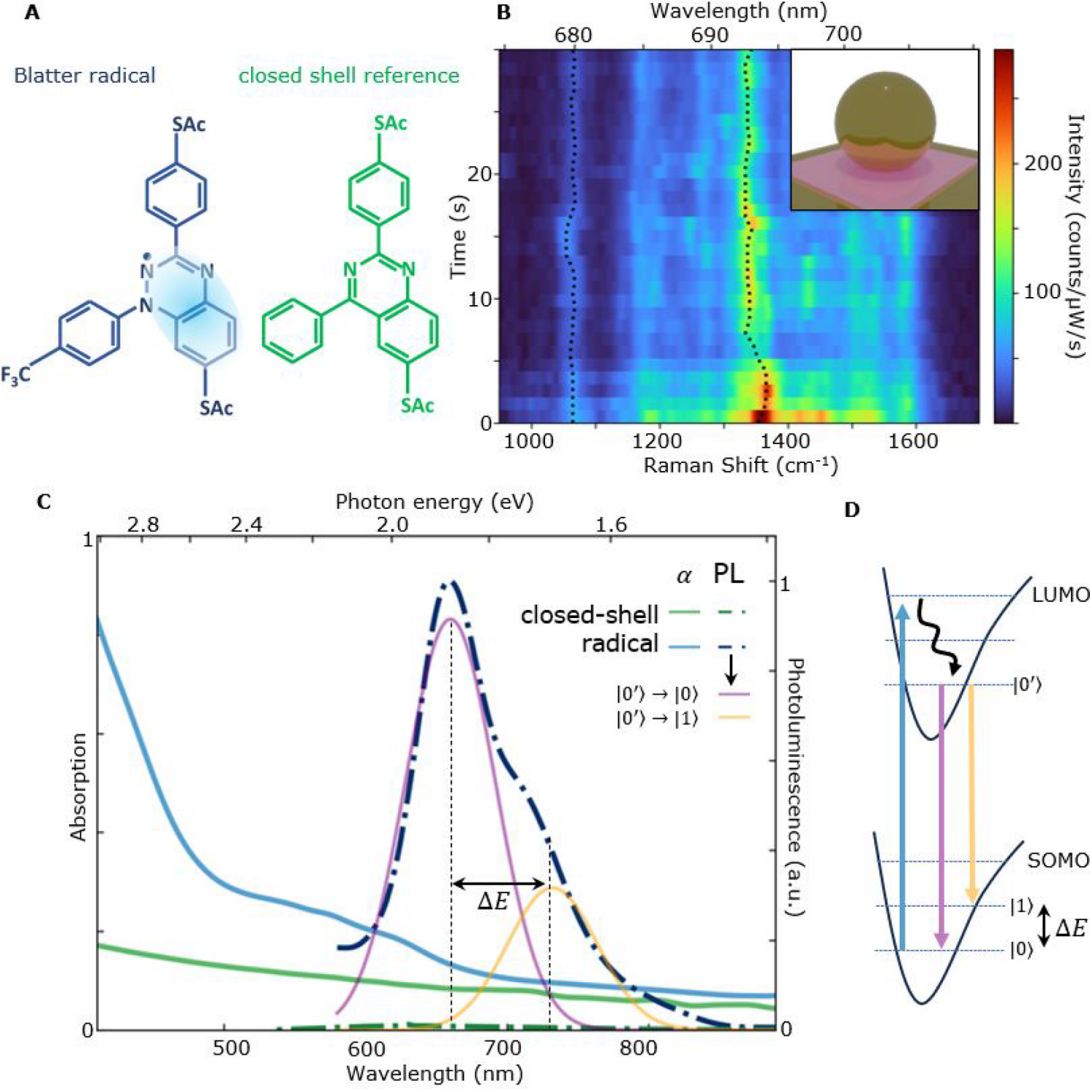
(A) Molecular structure of the Blatter radical and reference molecule with a closed shell (“closed-shell”). The standard acetyl [C(O)Me] protecting groups on the sulfur atoms are removed upon contact with gold.^21^ Delocalization of the radical electron over the conjugated core is shaded. (B) Time-resolved SERS spectra of *∼*200 Blatter radicals in a plasmonic nanoparticle-on-mirror (NPoM) system (illustrated in the inset). Dotted lines follow the maxima of the 1065 and 1360 cm^–1^ SERS lines, showing their energy variation. (C) Absorption (α, solid) and photoluminescence (PL, dashed) spectra for the radical (blue) and closed-shell reference (green) molecules in ethanol. The radical has a 40 meV smaller band gap but PL *∼*400× stronger. PL is decomposed into two Gaussians, from 0′ to 0 and 0′–1 decay to ground and excited vibrational levels, as in (D). Vibrational energy, Δ*E* ∼ 0.16 eV∼1360 cm^–1^, from the dominantly coupled molecular vibration.

A stable neutral organic radical of recent interest is the Blatter radical,^22^ with the stability attributed primarily to electron delocalization over several conjugated bonds around the molecule core.^23^ Recent developments of multiple variants to the original molecule synthesized by Blatter and Lukaszewski^22^ include addition of different head groups as well as a diradical system.^23−28^ Here, we investigate a stable doubly thiolated Blatter radical species and a closed-shell (nonradical) analogue (Figure 1A). The electron-withdrawing CF_3_ substituent further stabilizes the radical^29^ on a gold surface, and the double thiolation allows precision binding of both molecules to noble metals. The conductance and thermopower properties were previously examined in single-molecule junctions with a view to applications in thermoelectric devices, energy storage, and molecular electronics.^9^ However, knowledge of the optical properties as well as electronic and vibrational excitations of Blatter radicals remains limited.^25,30^

To access the molecular vibrational (Raman) spectrum of oriented single monolayers of these molecules, we incorporate them into gold nanoparticle-on-mirror (NPoM) plasmonic nanocavities^31−36^ (Figure 1B, inset). This construct is essential to confine light to the nanoscale and thus elicit signals from individual well-organized molecular self-assembled monolayers (SAMs).^37,38^ The surface-enhanced Raman scattering (SERS) from molecules in the gap between NPs and the mirror is enhanced by >10^9^ owing to the extreme light confinement.^31−33,39−41^ This allows for tracking the evolving time-varying SERS spectra of a target molecule (Figure 1B), which gives an improved understanding of its dynamics.

## Results/Discussion

### Electronic Excitations

The absorption and emission spectra of the thiolated radical and the closed-shell control are first compared in an ethanol solution (Figure 1C). The radical absorption switches on around 600 nm, as reported previously.^25^ This arises from the band gap between the singly occupied molecular orbital (SOMO) and the lowest unoccupied molecular orbital (LUMO) levels as calculated.^25^ The solvated radical gives PL in the visible and near-IR (NIR) region of the spectrum when excited by green light (at 532 nm). Its spectral line shape shows a shoulder beyond the main PL peak at 660 nm (1.9 eV) due to decay into the first vibrational excitation of the electronic ground state (Figure 1D). This energy spacing of 0.16 eV∼1360 cm^–1^ is in good agreement with the Raman spectra (Figure 1B, Figure 3A) as elaborated below. In comparison, the closed-shell reference molecule with a nearly identical structure but no unpaired electron (Figure 1A) shows very weak absorption and negligible emission in the visible regime due to the absence of the SOMO state in the closed shell. We note that strong luminescence from stable radicals is not common^16,17,42,43^ and extremely useful for quantum information applications since it opens the path to optical manipulation.^17,42,43^

### Molecular Monolayer Quality

**Figure 2:**
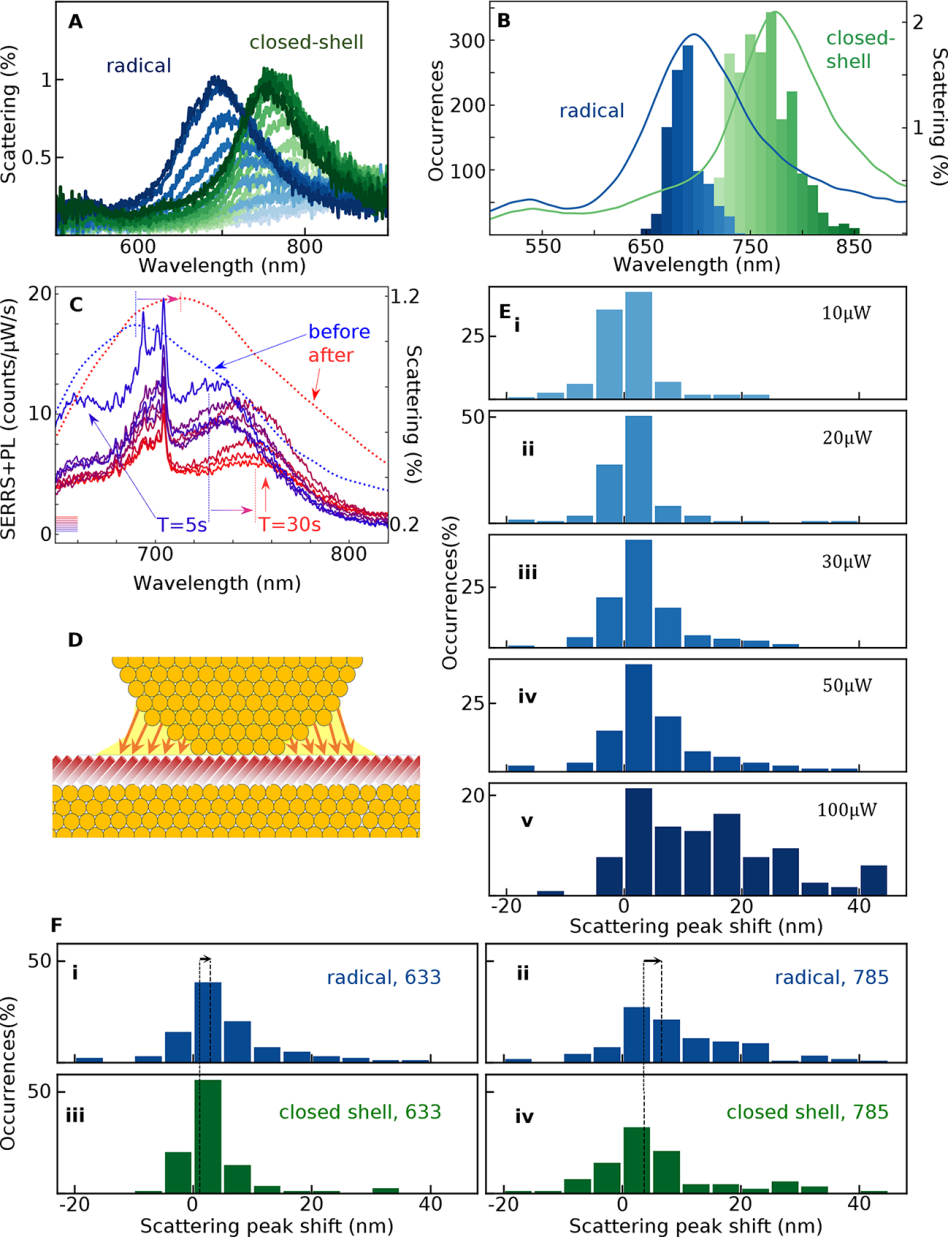
White-light scattering spectra of NPoMs. (A) Raw DF spectra for two NPoMs containing either the Blatter radical (blue) or the closed shell (green), constructed from focus-stacks to give a chromatically corrected scattering spectrum. (B) Histograms of the initial NPoM peak scattering wavelength showing 80 nm spectral shift between radical and closed-shell molecules. (C) Scattering spectra (dashed) from a single NPoM before (blue) and after (red) laser irradiation, overlaid on emission spectra [solid, from initial (blue) to final (red)], showing that both scattering and emission peaks redshift during irradiation. (D) Schematic of the bottom facet rearrangement (orange arrows) during irradiation. (E) Histograms of the scattering shift from before vs after laser irradiation for excitation powers from (i) 10 μW to (v) 100 μW. (F) Scattering shifts for radical vs closed-shell molecules for 50 μW irradiation at (i,iii) 633 nm and (ii,iv) 785 nm. Dashed line shows the average shift for each case (larger for the radical).

On template-stripped (TS) Au mirrors, SAMs of the radical and the closed-shell reference molecules can be straightforwardly laid down (see the Methods section). Both molecules are readily soluble in ethanol and form SAMs with short incubation times. To check the SAM quality, the white-light scattering spectra of many (>1000) individual NPoMs were measured across each sample using automated microscopy (see the Supporting Information, Figure S2). For each NPoM, an aberration-corrected darkfield (DF) scattering spectrum was recorded by combining spectra taken while scanning vertically through the focus (aFigure 2A). Histograms of peak scattering wavelength λ_m_ across all NPoMs with the Blatter radical or the closed-shell reference were then constructed (Figure 2B). The NPoM λ_m_ is set by the SAM thickness, refractive index, and nanoparticle geometry.^33−35,44−47^ Since both molecules give relatively narrow distributions of λ_m_, this implies that robust uniform SAMs are formed. The observed Δλ_m_∼ 80 nm difference between the histogram scattering peaks (for identical nanoparticle batches and such structurally similar molecules) cannot be explained by differences in electrical conductance (reported previously for these molecules^9^) as the shift should be opposite in sign.^48^ Ellipsometry (Figure S3) shows these SAMs are highly uniaxial and that the refractive index of the radical SAM is considerably higher than its control. Since the radical SAM is expected to be more sparse (compared with the closed-shell SAM) due to the bulkier electron-withdrawing CF_3_ group,^9^ the molecular polarizability of the radical molecule must then be much greater than that of the closed shell. However, this increased SAM refractive index would redshift the resonance (with respect to the closed-shell molecule), in contrast to what is observed for the DF resonance. Therefore, our data are only explained if the radical molecule is near-vertical to the surface while the closed-shell is tilted, making the radical SAM thicker and blueshifting its NPoM scattering spectrum with respect to the closed-shell molecule. Compared with other SAM layers in NPoMs,^33,46,49^ the thickness is estimated to be ∼1.7 nm, which is confirmed by the observation of PL since in-plane dipole emitters would be quenched in this NPoM geometry.

While it is not yet possible to directly probe the radical nature of the SAM, the data presented here including scattering and SERS spectra, refractive index measurements, and CV, alongside molecular electronic and thermal conductance measurements and DFT calculations published previously,^9^ provide strong evidence that the Blatter radical retains its radical nature in the SAM.

The emission from each radical-SAM NPoM when pumped by a 633 nm laser is made up of a combination of sharper SERS vibrational peaks and broad PL, which is enhanced by the plasmonic resonance (Figure 2C). Notably, the PL is composed of a single peak near that for 0–0 in solution, suggesting that the branching ratio is modified in the plasmonic environment (see below).

**Figure 3:**
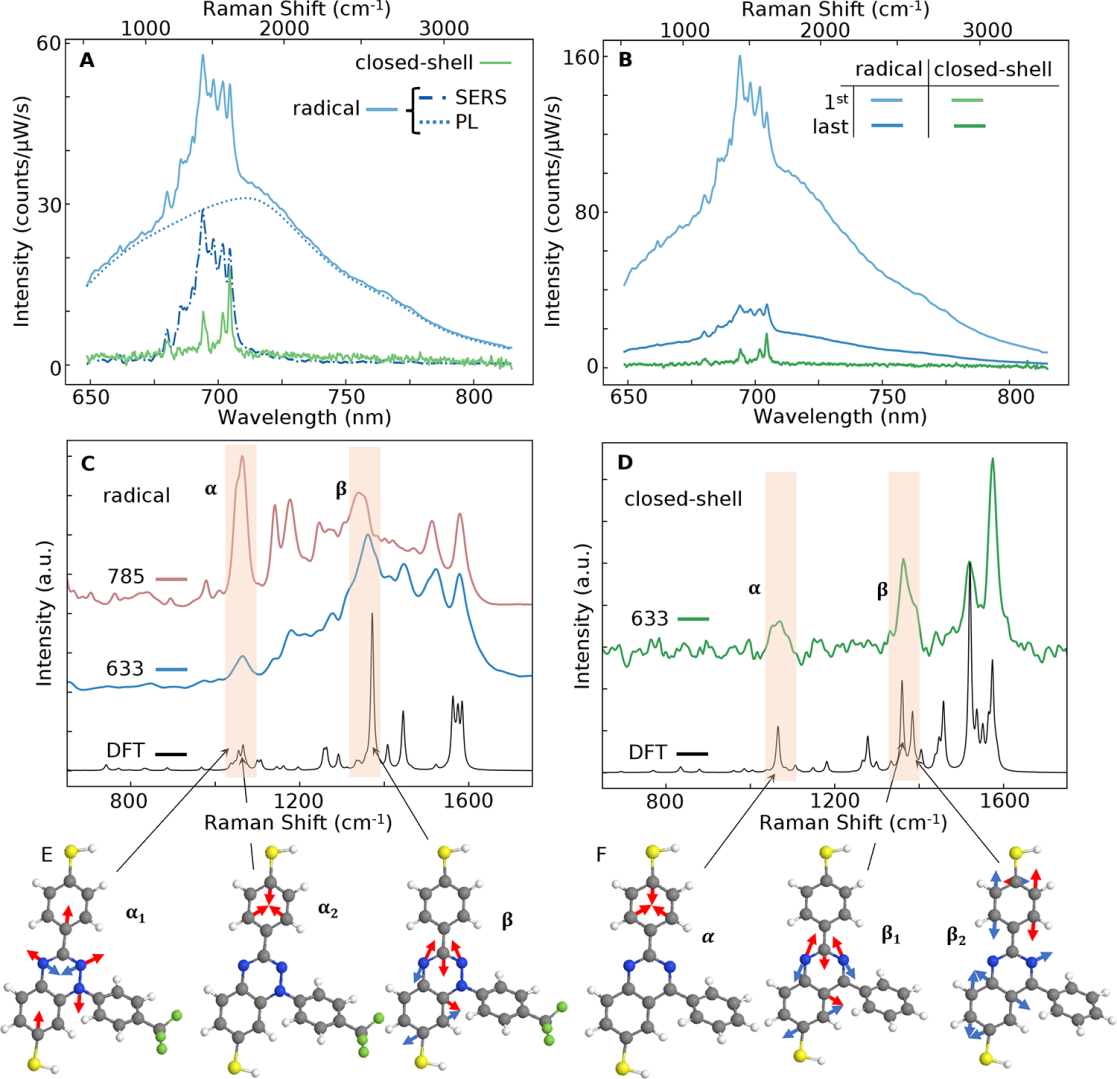
Average emission spectra of NPoMs. (A) Emission spectra of the Blatter radical (blue) composed of SERRS spectra (dashed) on a broad PL background (dotted), compared to the closed-shell molecule (green) without PL. (B) Average initial and final spectra of radical-filled NPoMs, showing PL bleaching over *T* = 30 s. For the closed-shell molecule, no bleaching is observed, and the initial and final spectra overlap exactly. (C) Comparison of SERRS peaks of the radical for 633 nm (blue) vs 785 nm (pink) excitation compared to DFT (black) calculation. The main vibrations shaded are 1065 (α), 1365 (β), and 1580 cm^–1^. (D) SERS spectrum of the closed-shell reference molecule (green) compared to the DFT calculation (black). (E,F) Illustrations of the α, β vibrational modes for the (E) Blatter radical and (F) closed-shell reference.

In most cases, the DF scattering peak redshifts during this laser irradiation (Figure 2C–F). Comparing this NPoM DF redshift to emission spectra over *T* = 0–30 s irradiation time (Figure 2C) shows both redshift by similar amounts (here ∼30 nm), while the sharp SERS peaks decrease. Comparing histograms of these redshifts for many NPoMs (Figure 3E,i–v) for increasing laser powers up to 100 μW shows how the average redshift increases and the distribution broadens. This confirms that irreversible changes are light-induced above a threshold of ∼30 μW, as previously seen for closed-shell SAMs.^50^ Below 30 μW, the redshift is minimal (Figure 2E,i–iii). The average redshift is consistently larger for the radical SAM than for the closed-shell analogue (Figure 2F), and it is larger when the NPoMs are excited by 785 nm compared with 633 nm, despite the latter exciting the molecular transition (Figure 2F). This redshift of the plasmonic resonance is a result of a light-induced resculpting and widening of the NPoM bottom facet (Figure 2D), which gives redshifts.^46,47^ Even when excited somewhat off-resonance, the electronic state of the radicals gives higher polarizability^31,36,41^ (due to the HOMO–SOMO transition, Figure 2C), leading to optical annealing of the facet from enhanced Au surface atom mobility.^51,52^ This mechanism is reported in several different molecular systems^31,36,41,46,47,51,52^ and is supported by a model^41^ based on light-induced van der Waals attraction of Au that scales as the molecular polarizability. Our results agree well with previous reports and the model, having observed significant plasmonic redshifting for the highly polarizable radical molecule and significantly lower redshifting for the similar but less polarizable closed-shell reference.

### Surface-Enhanced Resonant Raman Spectrum

Because the laser is near-resonant with the radical HOMO–SOMO energy, its emission gives surface-enhanced resonant Raman scattering (SERRS), enhancing the vibrational peaks that are coupled to the electronic transition. A comparison of the average SERRS spectrum of the Blatter radical SAM (Figure 3A, blue) and the closed-shell reference (green) for 633 nm pumping shows that the former includes a broad PL peak centered around 700 nm (blue dotted), slightly red-shifted from the solution PL and matching the DF scattering resonance λ_m_∼ 700 nm. Asymmetric least-squares (ALS)^53^ fitting enables separation of the PL (dotted) and SERRS peaks. Over time, the radical PL decreases due to bleaching, which also decreases the SERRS resonant enhancement (Figure 3B).

While the closed shell exhibits four distinct peaks (α, β, and two additional peaks at ∼1550 and 1590 cm^–1^), the radical molecule displays additional overlapping and broad SERRS peaks from 1000 to 1600 cm^–1^. These correspond mainly to stretching modes of the molecule’s conjugated core, as calculated by density functional theory (DFT, Figure 3E,F); see the Computational Method section for details of DFT calculations. Importantly, the wavenumber peak at 1365 cm^–1^ (labeled β) matches the PL vibronic sideband seen in the PL above (Figure 1). For the radical molecule, the SERS spectrum closely resembles the Raman spectrum recorded on crystalline powder (see the Supporting Information Figures S5 and S6). During laser irradiation (Figure 3B), the β vibrational peak weakens due to a combination of redshifting of the plasmonic resonance (shown above by white-light scattering), as well as due to quenching of the electronic transition, which initially enhances the vibrational scattering. A comparison of the SERS for 633 and 785 nm excitation with the DFT-calculated spectra (Figure 3B) for the radical SAM shows the selective enhancement of different vibrations. In particular, the 1065 cm^–1^ line (labeled α) is strongly enhanced for the near-resonant 785 nm excitation. It is evident that the measured and DFT-calculated spectra for the closed-shell nonresonant molecule (Figure 3D) match peak ratios better than the radical molecule. These peak ratios do not vary significantly as the radical PL bleaches (Figure 3B), suggesting that some molecules never bleach. As previously found, this can arise due to the Purcell-enhanced speed-up of emission, which reduces the probability of singlet–triplet crossing and subsequent reactions.^54^

The key difference in emission from radical and closed-shell molecules is the visible PL together with the broad vibrational band (discussed further below). Both of these originate from the delocalized orbitals of the radical in the molecular core, which originate from orbitals occupied by the unpaired electron, as shown by DFT. Both the PL and broad vibrational band are thus closely related to the radical nature of this molecule and can be potentially used to probe the radical dynamics in precision nanoscale environments.

### PL Decay

**Figure 4:**
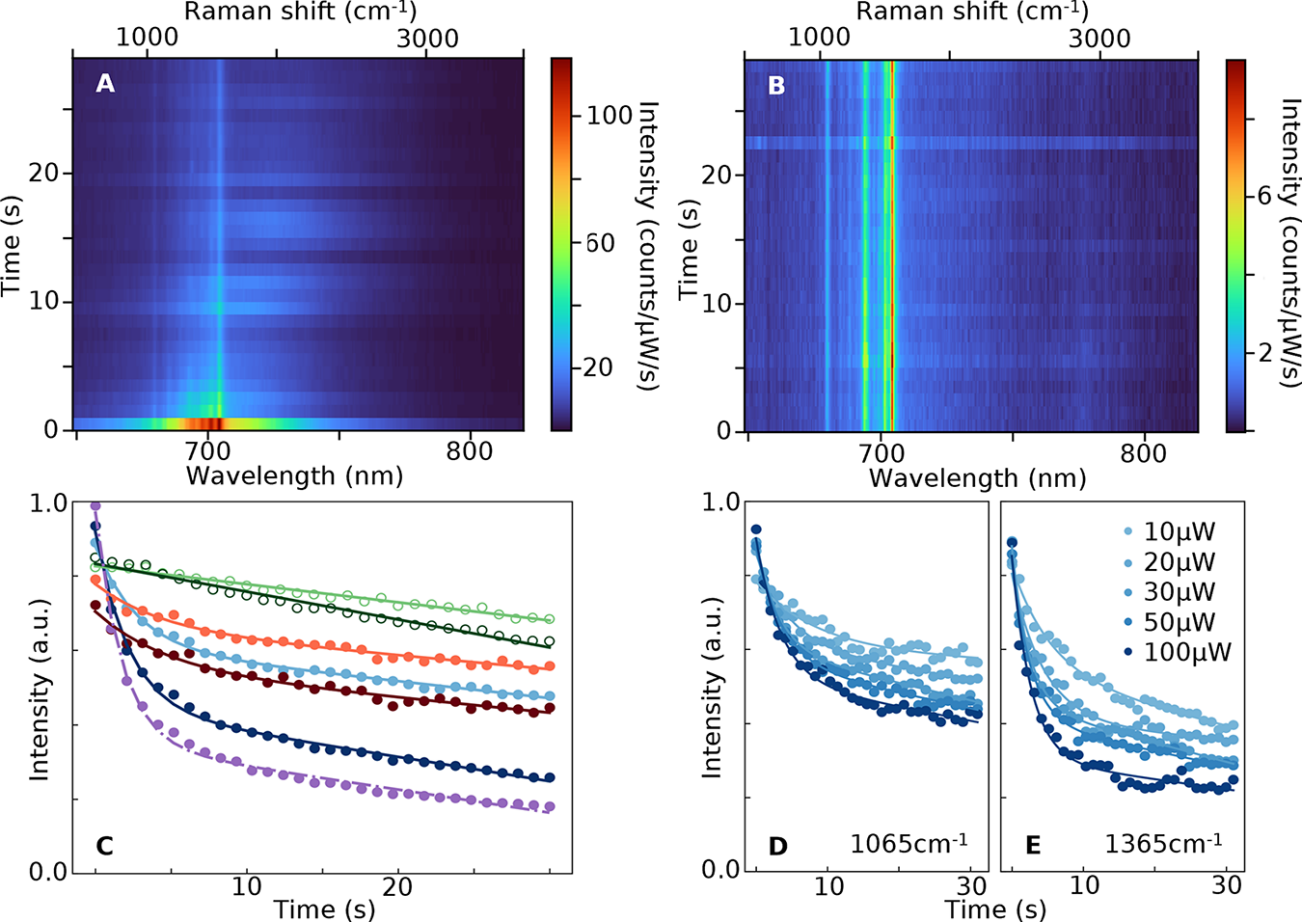
SERRS decay. (A) Time-dependent emission from an NPoM with the Blatter radical SAM, showing fast PL quenching in ∼2 s. (B) Time-dependent emission from NPoM with closed-shell SAM. (C) Average temporal evolution of 1065 (light) and 1365 cm^–1^ (dark) SERRS lines for the Blatter radical with 633 nm (blue) and 785 nm (red) pumps for closed-shell (green circles) and PL (purple). Fits are to a sum of fast exponential and slow linear decay: *I*(*t*) = *A*exp{−*t*/τ_e_} – *Bt*/τ_L_ + *C* (radical) or linear only (closed-shell) decay. (D,E) Evolution of radical (D) 1065 and (E) 1365 cm^–1^ SERS peaks at increasing powers (10–100 μW, 633 nm).

The radical molecule emission shows a rapid intensity decay, with both PL and SERS decreasing within seconds after the initial laser irradiation (Figure 4). In comparison, the closed-shell species shows a stable (but much weaker) SERS and no decay (Figure 4B), confirming its lack of electronic transitions in the visible regime.

The decay of SERS peaks is well fitted to a sum of a fast exponential and a slower linear decay (Figure 4C), with time scales of τ_e_∼ 5 s and τ_L_∼ 200 s (respectively) seen in both α and β Raman lines as well as in the extracted PL. This decay is irreversible. Typically, 30–50% of the initial SERRS and PL intensity remain after 30 s, for excitation at both 633 nm (blue) and 785 nm (red). This corresponds to a set of molecules (in the center of the nanocavity field profile) whose decay is strongly sped up, that thus avoid bleaching.^54^ By comparison, the closed-shell SERS linearly decays by <10%, comparable to similar data from other nonresonant molecules, likely due to changes in facet morphology rather than bleaching (also matching the slow decay seen with the resonant radical).^33,40,55^ The fraction of bleached signal from radicals increases from 40 to 60% as the laser power is increased from 10 to 100 μW (Figure 4D), while the exponential decay time more than halves, from 7 → 3 s (for α) and 5 → 2 s (for β).

### SERS Line Jitter

Vibrational energies of small molecules in Raman spectroscopy are usually time-stable;^33,36,40,55−58^ however, the evolving SERRS peaks from Blatter radical SAMs show shifts on a subsecond time scale (Figure 1B). Time-averaging these peaks gives the broad vibronic features seen in the averaged SERS spectra (Figure 3A–D).

**Figure 5:**
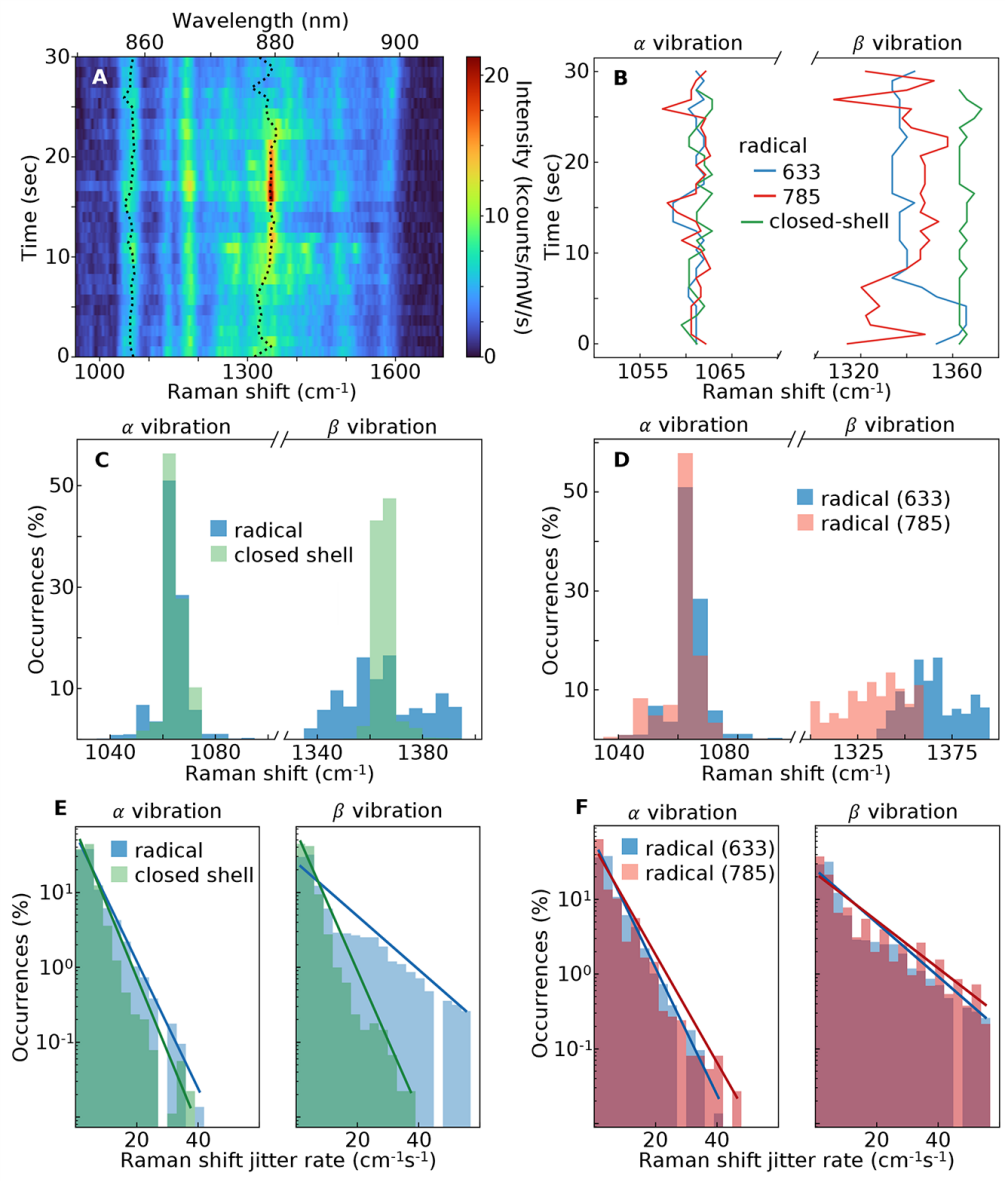
Time-jitter of Blatter radical SERS lines. (A) Evolving SERS of the Blatter radical in NPoM (785 nm pump), showing jitter of α and β peaks (dashed). (B) Extracted jittering of SERS peaks for three individual NPoMs, comparing radical and closed-shell SAMs at different laser λ indicated. (C,D) Histograms of SERS ν_pk_ for radical (blue) vs closed shell (green, C) and radical at 633 vs 785 nm (D). (E,F) Histograms of jitter rate, showing faster jitter for β vibration of the radical.

The frequency jitter in time of the SERS peaks from a single NPoM (Figure 5A) shows the instability of the α and β vibrations (dashed lines). In particular, β, which is a stretch vibration of the molecular core, shifts randomly without any noticeable long-term trend. Comparing the tracks from three individual NPoMs (Figure 5B) containing either a radical or closed-shell SAM and excited by different laser wavelengths shows significantly more jitter from the β than the α lines. This β line jitter is much lower for the closed-shell species. These properties are even more apparent from the histograms of peak wavenumber ν_*i*_(*t*) (Figure 5C,D). While the histogram widths of α are similar for the radical and closed-shell species, the radical jitter of β is so significant that it is hard to continuously identify the peak. This broadens the width of the average SERS spectrum (Figure 3) to ∼100 cm^–1^ (FWHM).

The jitter rate can also be extracted from the shift in peak Δν = |ν_*i*_(*t* + Δ*t*) – ν_*i*_(*t*)|, and plotting this histogram of Δν shows the probability of different frequency jumps (Figure 5E,F). We find an exponential decaying probability of larger jitter rates, *p*(Δν) ∝ exp {−Δν/*R*}, giving an extracted average jitter rate *R* (cm^–1^ s^–1^, Table 1). The jitter rates for the radical SAM are very significant, 3–8 times faster than reported for changes in molecular redox states^59^ or adatom interactions (“picocavities”).^40,60^ Since the rates *R*_β_ are ∼3-fold larger for the radical than the closed-shell species (independent of laser wavelength), this suggests that the vibrational jitter stems from the radical nature of the molecule.

**Table 1:** Average Jitter Rate Extracted from Histograms of Jitter Rates (in Figure 5E,F), in cm^–1^s^–1^

molecule	excitation (nm)	α average jitter rate (cm^–1^ s^–1^)	β average jitter rate (cm^–1^ s^–1^)
closed-shell	633	10 ± 1	11.1 ± 0.8
radical	633	12.5 ± 0.5	25 ± 1.3
radical	785	14.3 ± 1	33 ± 3

To check if transient interactions between neighboring radicals might be responsible for the observed jitter, the Blatter SAM was diluted (by up to 90%) with a closed-shell mercapto-biphenylcarbonitrile (BPTCN) molecule, which mixes well (Suppl. Info. Figure S7). Since the jitter in SERS of these more separated radicals is unchanged, the origin of jitter from spin-dependent electron interactions is not strongly supported (although DFT suggests it may be significant for very close separations, see Figures S10 and S11). Another possibility is the spin–orbit-like coupling between vibrational deformations and electron spin orientation or redox processes.

### Effect of Redox on Vibrational and Electronic Excited States

**Figure 6:**
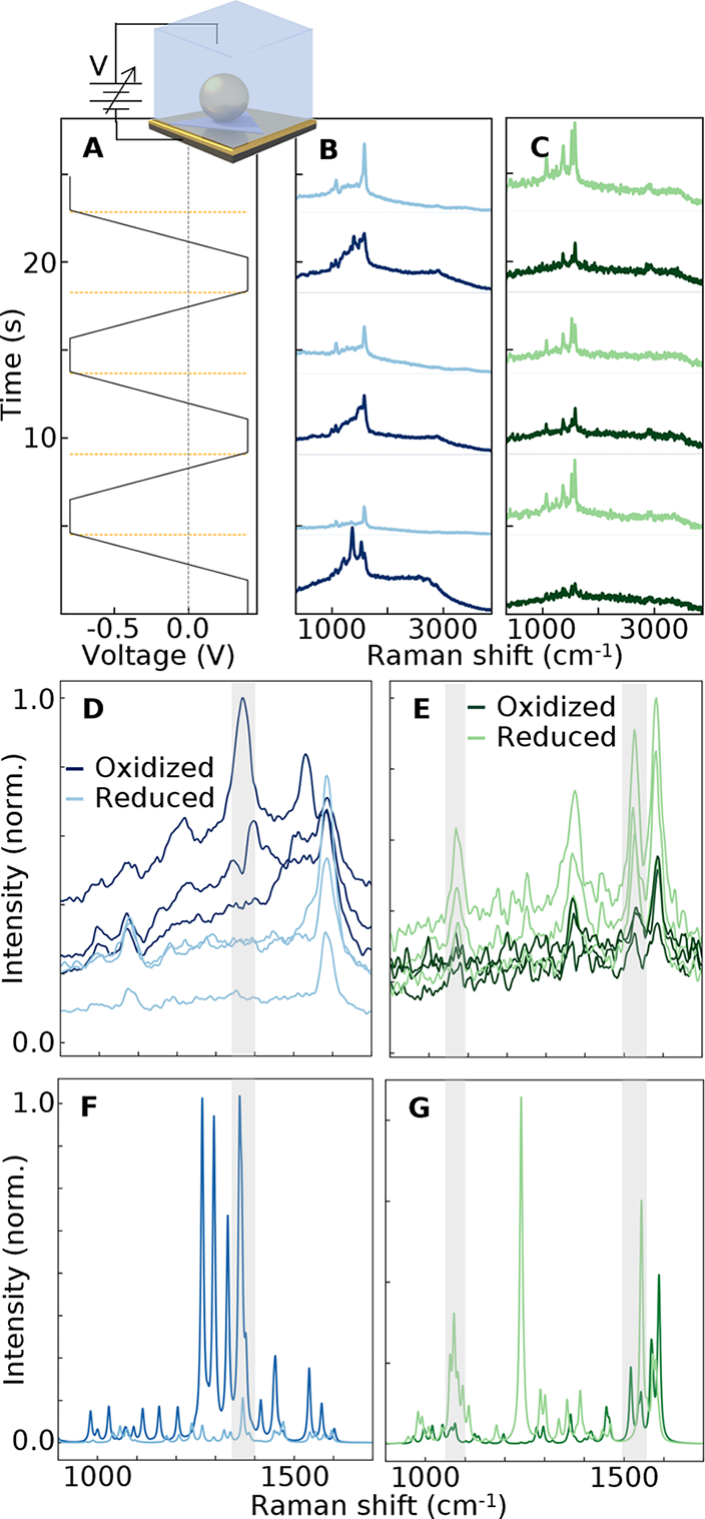
Measured SERS and PL for different potential steps. (A) Voltage steps applied to the Au mirror working electrode (inset). (B,C) Average spectra at each potential for radical and closed-shell SAMs, expanded to the range of interest in (D,E). (F,G) DFT-calculated SERS spectra for the (F) radical and (G) closed shell in oxidized and reduced states.

Cyclic voltammetry (CV) experiments were thus performed to investigate whether electron transfer between the metal facets and the molecules can influence both the PL decay and SERS jitter.^58,59^ Previous reports^8,23,61^ identified degradation over an extended time, which was suspected to arise from oxidation^23^ (although other explanations are possible^62^), while reversible redox processes were measured in electrochemical cells.^8,61^ Here, we observe improved redox reversibility when these radicals are presented as SAMs. After establishing a reduction potential in CV of ∼−0.9 V, the oxidation current peak is found to be not reached at +0.5 V (Supp. Info. Figure S8) and thus the applied potential was limited to between +0.4 and −0.8 V, which is however still sufficient to observe oxidation/reduction in SERS under the NPoMs.^23,63^ An underlying gold mirror was used as the working electrode in a purpose-designed electrochemical cell with 0.1 M sodium nitrate (NaNO_3_, aq.) electrolyte,^64^ and the potential switched from high (+0.4 V) to low (−0.8 V) while simultaneously measuring the SERS spectra over several cycles (Figure 6).

For the closed-shell species (Figure 6C), applying a positive potential significantly reduces the SERS intensity, which is regained for the negative potential. This resembles the SERS intensity changes seen for other thiols^65^ and originates from the reduced polarizability of the molecule under high electric fields in the NPoM gap.^65^ On the other hand, a positive potential enhances the SERS and PL from the radical (which correspondingly decrease at a negative potential, Figure 6B). In particular, at −0.8 V, the PL drops and the broad β vibration regime around 1360 cm^–1^ vanishes. As shown in Figure 1, the β vibration is enhanced by electronic resonance (resonant Raman, i.e., SERRS), and this seems to be quenched by reduction. Evidently, this process has a stronger effect on the PL\SERS emission than the change in molecular polarizability seen for the closed-shell molecule. In successive cycles, the PL gradually disappears as already noted above. DFT simulations (Figure 6F,G) support this reversed behavior for the radical and closed-shell species at opposite potentials, corresponding to the oxidized and reduced states. Illumination-induced PL quenching (and subsequent vanishing of the β peaks) makes further interpretations difficult. However, it is still clear that at a positive voltage (+0.4 V) the molecule returns to its neutral state, and the PL is restored along with the jittering β vibration (Figure 6D).

## Conclusions

The results above suggest that the unpaired radical electron on the Blatter molecule has considerable interaction with the molecular vibrations. This can be seen already in the additional electronic SOMO–LUMO resonance of the molecule (compared to the closed-shell reference), which leads to enhanced polarizability, which strongly increases the optical-induced forces that resculpt the neighboring Au facets as well as the enhanced SERRS from selected vibrations. Literature suggests this coupling can be a result of the molecular conformation slightly changing in response to the electric field applied by the unpaired electron.^66^ In particular, we find that one of the ring vibrations that spatially overlaps the radical electron strongly varies in vibrational frequency over time; however, the exact conformational changes cannot yet be distinguished from the spectra that jitter as this is not yet computationally tractable. This jitter is on relatively slow time scales compared to the expected dynamic spin variation of the radical, and this is not found to change in applied magnetic fields up to 40 mT. Electrochemical step measurements suggest the same vibrations are also sensitive to redox processes, and thus fluctuations in the position of the radical electron may also be responsible. The mechanism of light-induced PL- (and corresponding SERS-) quenching is not fully identified^54^ but typically is ascribed to chemical oxidation, which has been improved through suitable encapsulation. Another possible explanation for the irreversible quenching is a conformational change in the molecule (possibly tilting) that shrinks the SAM thickness, thus redshifting the plasmonic resonance and providing an in-plane component to the molecular dipole that is rapidly quenched by the metal.

The ability to measure and address few (or even single) spins at room temperature under ambient conditions is of considerable interest for quantum information applications, as well as local sensing. The Blatter radical system coupled with advanced plasmonic nanocavities is thus a highly promising and versatile platform for further fundamental studies and for the development of a wide variety of potential applications.

## Methods/Experimental

Molecular synthesis has been reported in ref (9). The diazine closed-shell molecule is chosen as a reference molecule with a similar structure since a triazine structure with an *N*-phenyl substituent would be cationic (due to the quaternary N atom) and would thus present a larger perturbation of the system, compared to replacing N with C (triazine radical; diazine neutral) as chosen here.

### Solution Formation

Blatter radical and closed-shell reference molecules were dissolved in anhydrous ethanol at a concentration of 1 mM. Figure S1 shows the radical nature is retained in solution. The solution was sonicated for a few minutes until no powder crystals were identified visually. The solution (radical in solvent) was wrapped in aluminum foil to avoid light exposure and kept refrigerated at 4 °C.

### TS Gold Films

The films were produced by thermally evaporating a 100 nm Au film on a Si (100) wafer. Small glass slides (0.5 × 5 × 10 mm, UQG optics) were glued by UV adhesive (Norland 81) onto the Au layer and cured for 30 min under a UV lamp.

### Sample Fabrication

Gold-coated glass substrates were pulled off the silicon wafer and immediately placed in the molecular solution for 20 min. After the samples were taken out of the solution, they were thoroughly rinsed in ∼50 mL of EtOH and blow-dried in nitrogen; 30 μL of Au nanoparticle solution (80 or 100 nm, BBI, nanoparticles washed twice with DI water to remove excess citrate) was drop-cast on top. To this nanoparticle solution was injected 5 μL of NaNO_3_ 0.1 M solution to ensure adhesion to the metal film. After 10 s settling time, the sample was washed with DI water and blown dry with dry nitrogen. Samples were measured within 2–3 h after fabrication. However, samples up to a few weeks old did not show significant effects of aging and still showed typical SERS spectra after months.

### NPoM Scattering and SERS Spectra

The scattering and SERS spectra of NPoMs were collected using a home-built semiautomated microscope (sketch in the Supporting Information Figure S2) based on an Olympus BX60 microscope with a 100× DF objective lens, capable of measuring both white-light scattering spectra (using an OceanOptics QEpro spectrometer) and SERS using an EM-CCD camera (Andor-Newton) coupled to a monochromator (Andor-Kymera). The sample was placed on an automated stage (Prior Scientific). The microscope illumination arm was used to illuminate the sample for both identifying individual NPoMs (through scattering images) and providing a light source for scattering spectra. For scattering, multiple spectra were taken at different sample heights to correct for the objective chromatic aberration. The SERS signal was calibrated by measuring the Raman spectra of silicon wafers, with a typical efficiency of 4500 counts/mW/s for the silicon Stokes–Raman line at 520.6 cm^–1^. The Raman shifts were calibrated by measuring the Stokes and anti-Stokes Raman peaks of silicon, which allow identification of the exact laser wavelength. The SERS excitation sources were single-frequency diode lasers at 633 nm (Matchbox, Integrated Optics) and 785 nm (FPV-785S, Thorlabs). During measurements, camera images were used to exclude from analysis nanoparticle aggregates (much brighter, wrong shape) in real time, and postselection was used to filter out SAM and mirror defects. Postselection primarily uses the recorded images, as well as selects only DF spectra with a peak beyond 550 nm. We see from the average scattering spectrum that this does not introduce artifacts or skew the results, as >98% of the remaining spectra have a peak above 650 nm.

Electrochemical measurements were performed using a low-profile home-built electrochemical cell, which fits below the objective lens of our optical system. The TS-Au sample was used as the working electrode, Pt wire as the counter electrode, and Ag/AgCl electrode as the reference. Voltage and current signals were recorded by using an Ivium-Vertex potentiostat. The electrolyte was a 0.1 M aqueous solution of sodium nitrate (NaNO_3_). The scan rate was 0.1 V/s for the radical and 0.05 V/s for the closed-shell molecule.

Liquid absorption measurements were performed with a home-built setup based on a fiber-coupled light source and Ocean Optics QEpro spectrometer and also for reference with an Agilent Cary100 UV–vis spectrophotometer.

PL measurements were performed using both a Renishaw InVia Raman microscope (using laser excitation) and an Agilent Cary Eclipse Fluorescence spectrometer (with an excitation spectral width of ∼10 nm).

Data analysis was performed in Python using built-in packages. The SERS background was removed using an ALS (Whittaker) algorithm^53^ to fit and subtract a polynomial of high degree. Several preprocessing steps were taken to ensure the fitted background was below the signal.

### Computational Studies

The ground-state geometries of the molecules in the absence and presence of gold electrodes were obtained after performing geometry optimization using the Gaussian g16^67^ implementation of DFT. B3LYP hybrid functionals with the QZVP basis set and tight convergence criteria were used with a quadratically convergent SCF procedure. We then calculated the Raman spectra for molecules with different configurations, as discussed in the main text and in the SI.

## Supporting Information Available

Results of EPR spectroscopy of the radical, detailed description of the automated optical setup, measurement of the SAM refractive index, powder Raman spectroscopy of molecules, measurements of mixed SAMs, and DFT calculations of the molecules’ SERS cross section in redox states and when dimerized (PDF)
