# Supplementary material for: Electronically Perturbed Vibrational Excitations of the Luminescing Stable Blatter Radical
Source: ACS Nano. 2025 Feb 21;19(8):7650–60. doi: 10.1021/acsnano.4c09661 (PMC11887450; doi:10.1021/acsnano.4c09661)
Supplement: Supplementary file 1 — nn4c09661_si_001.pdf [file nn4c09661_si_001.pdf]

Supplementary information for: Electronically-perturbed vibrational excitations of the luminescing stable Blatter radical

Jonathan Bar-David[1], Abdalghani Daaoub[2], Shangzhi Chen[1], Sarah May Sibug-Torres[1], Sara Rocchetti[1], Gyeongwon Kang[1], Ross J. Davidson[3], Rebecca J. Salthouse[3], Chenyang Guo[1], Niclas Sven Mueller[1], Sara Sangtarash[2], Martin R. Bryce[3], Hatef Sadeghi[2*], and Jeremy J. Baumberg[1*]*

[1]NanoPhotonics Centre, Cavendish Laboratory, Dept. of Physics, University of Cambridge, Cambridge, CB3 0HE, UK

[2]Device Modelling Group, School of Engineering, University of Warwick, Coventry CV4 7AL, UK

[3]Dept. of Chemistry, Durham University, Durham, DH1 3LE, UK

[*]emails: jjb12@cam.ac.uk, hatef.sadeghi@warwick.ac.uk, m.r.bryce@durham.ac.uk

EPR of Blatter radical in ethanol

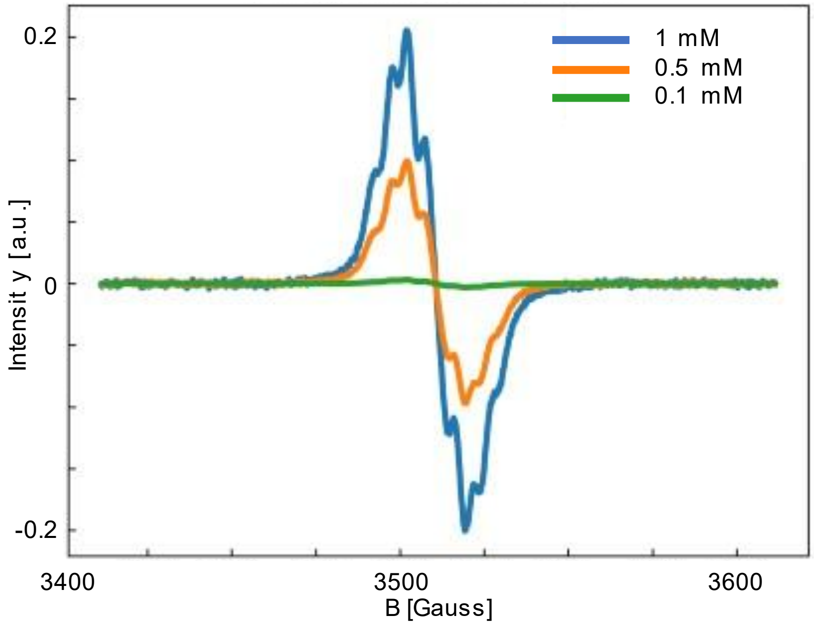

Figure S1. EPR measurement of Blatter radical in solution showing the singlet nature of the radical and the sublevels associated with electron interaction with the three nitrogen atoms.

Automated microscope system

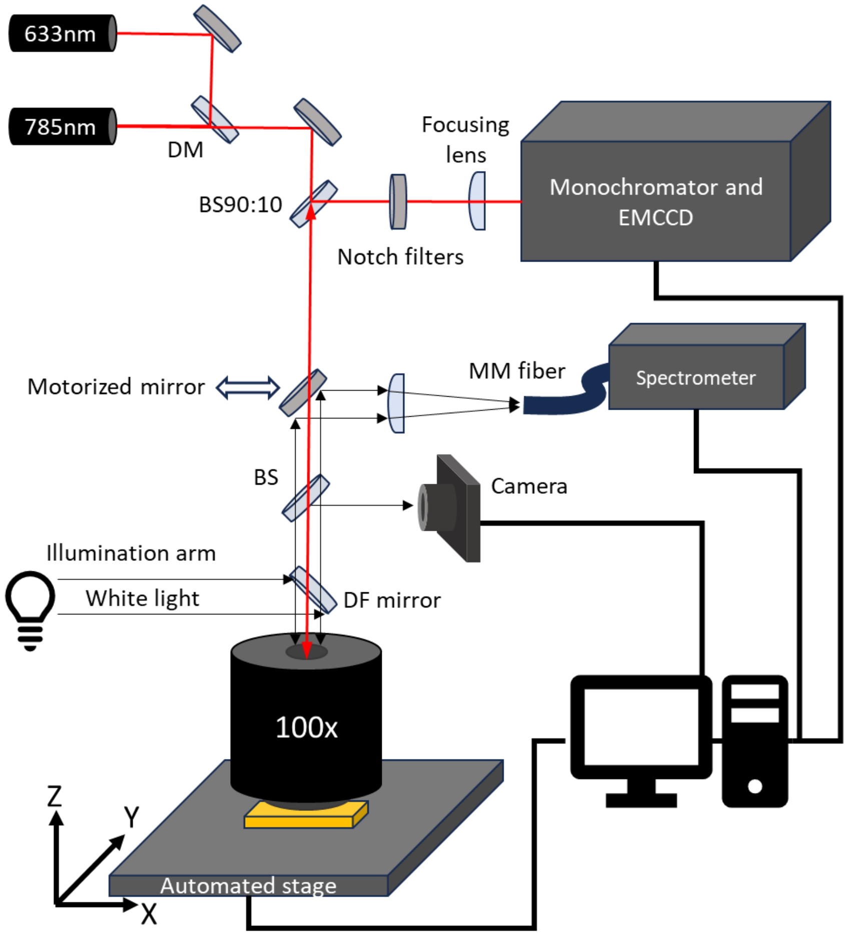

Figure S2. Automated microscope system. The 633 and 785 nm excitation lasers are combined at a dichroic mirror (DM) and aligned into the 100x 0.9NA darkfield microscope objective. In the optical path several beamsplitters (BS) are situated to allow imaging (by CCD camera), white light scattering (by fiber-coupled spectrometer) and SERS measurement (through 90:10 beamsplitter). The laser line is filtered by spectral notch filters and focused onto the entrance slit of a monochromator coupled to an EM-CCD camera. All moving parts and instrumentation, including motorized microscope stage, are computer controlled.

SAM quality and refractive index

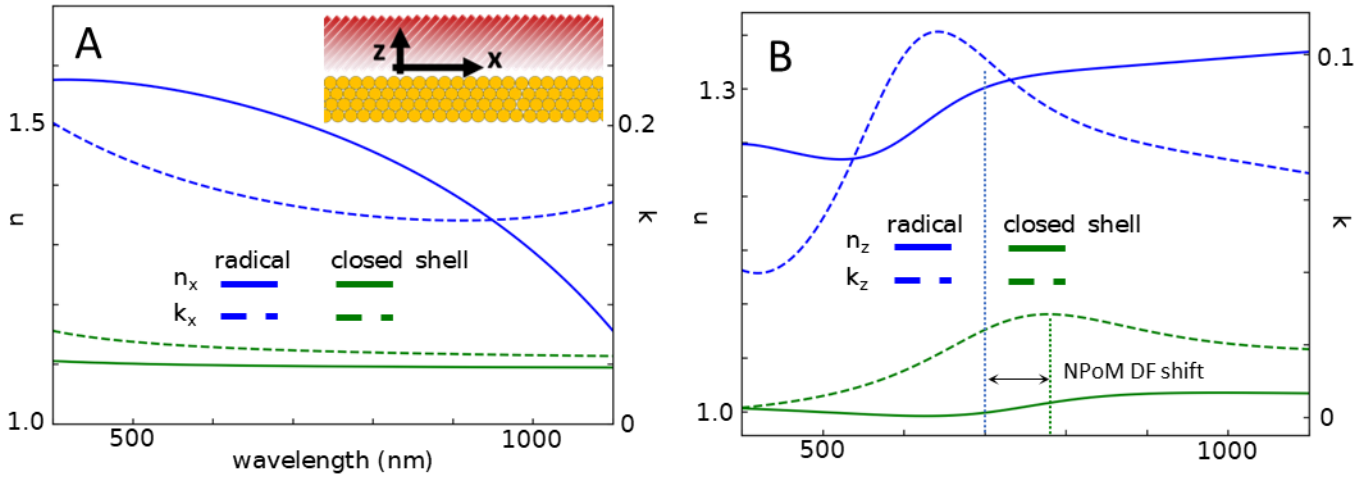

Figure S3. Experimental n,k values from ellipsometry for both (A) in-plane (x) and (B) out-of-plane (z) components of radical and closed-shell SAMs. SAMs are highly reproducible. Model fits show a ~0.4 refractive index change (from energy shifts) and a thickness difference from 2.45nm for radical to 2.6nm for closed-shell molecule. inset: definition of in-plane (ordinary) and out-of-plane (extraordinary) field components in SAM.

The refractive index of both radical and closed shell SAMs are measured by ellipsometry. Apart from results being highly reproducible across different samples, the fitting model (in figure S3) suggests that the refractive index of the radical molecule (fig, S3 A,B, blue curve) is higher than that of the closed shell molecule, and also has a strong resonance (absorption band) at ~650nm, generally matching the scattering spectrum of NPoM constructs and, clearly is at a shorter wavelength relative to the (weak) resonance of the closed shell molecule. The results of refractive index measurements combined with the consistent SERS are evidence for the existence of well-formed SAMs on these samples.

Irreversibility of scattering peak shift

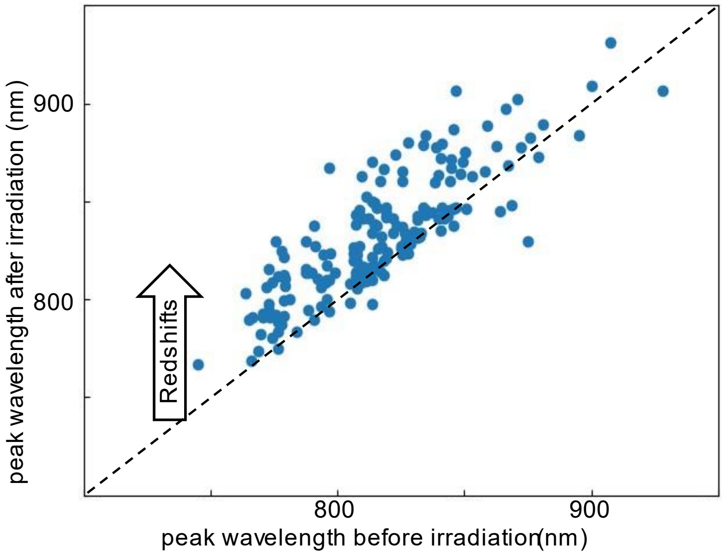

Figure S4. Scattering peak wavelengths of 100nm NPoMs irradiated by 785nm laser before and after irradiation. Power is gradually increased from 10 to 100µW and decreased back to 10µW. The resonances permanently shift and do not recover at low irradiation power.

SERS and PL of molecule crystal powder

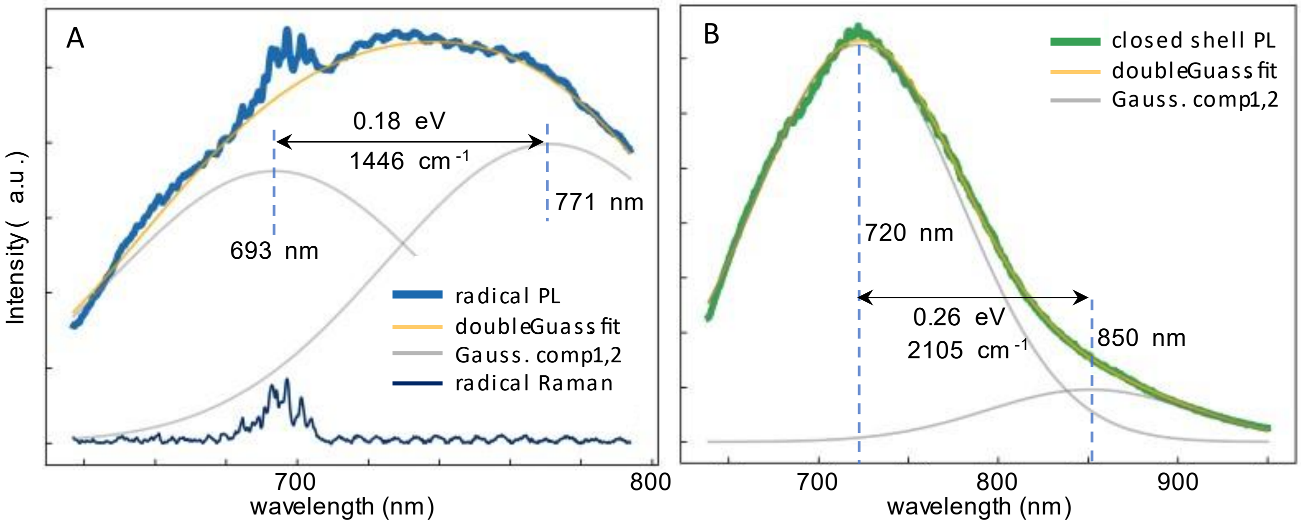

Figure S5. Raman spectrum on dry powder. A) radical and B) closed shell molecule. Both molecules show photoluminescence. For the closed shell molecule, the Raman signal is completely lost in the PL background.

Figure S5 shows the recorded Raman spectrum from molecular crystal powder. The radical molecule exhibits a Raman spectrum with a broad PL background which we identify as originating from decays into an electronic ground state (693 nm) and a significant vibronic band (771 nm) with vibrational energy of 0.18 eV or 1446 cm^{-1}. The closed-shell molecule exhibits a main PL peak at 720 nm with a small vibronic band at 2105 cm^{-1}, but the Raman spectrum is not identified on this strong background. The PL of the closed shell molecule was not seen in solution or in the SAM. While not investigated, this might be a result of the crystal structure of this molecular crystal, as reported for other fluorophores[1,2]. In figure S6 we present the extracted Raman spectrum of the radical molecule, compared with SERS and DFT spectrum presented in the main text. The Raman, DFT calculation and SERS spectra match well, with both vibronic bands (1365 cm^{-1}, identified in solution PL, and 1446 cm^{-1}, identified in the powder PL) present in both spectra.

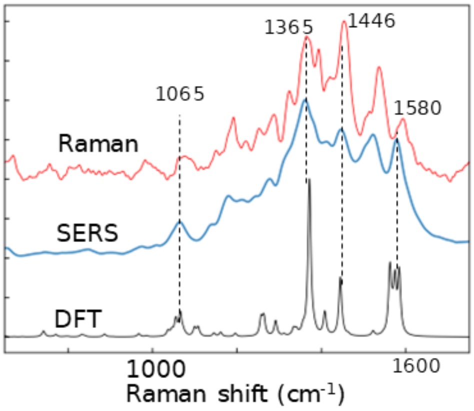

Figure S6. Powder Raman spectrum of the radical molecule aligns well with the SERS spectrum and DFT calculation reported in the main text.

Electron-electron coupling as source of SERS line time-jitter

To examine whether electron-electron coupling is the source of the time-jitter observed for radical SAMs we examined the SERS spectra for mixed SAMs of Blatter radical and mercapto-biphenylcarbonitrile (BPTCN) in a molar ratio of 1:10. We established a baseline for BPTCN SERS by measuring a pure BPTCN SAM, the average spectrum of which is presented in Figure S7(A). BPTCN was chosen due to its stable and strong SERS signal, with a distinct $\mathrm{C} \equiv \mathrm{N}$ stretch (Figure S7A, inset) producing a Raman line ~2200 cm^{-1}. Principal component analysis (PCA) was used to decompose the data into two components (Figure S7B). After measuring SERS of mixed SAMs (Figure S7Ci), the data was reconstructed with the BPTCN components (Figure S7Cii) and this reconstruction was subtracted from the original data (Figure S7Ciii). Figure S7D shows the extracted radical spectra. The α and β vibrations are indicated by dotted pink lines, and their time-jitter is easily noticeable. Figure S7E shows the histogram of peak locations for the α and β vibrations (blue), compared to BPTCN lines (green). It is evident from the histogram that the radical jitter is larger compared with the more stable BPTCN. Figure S7F shows the time jitter rate, with an average jitter rate of 20-25 $cm^{-1}s^{-1}$ which is very similar to our results for pure radical SAMs. Therefore, we consider it unlikely that the source of the evolving jitter in the SERS spectra of radical SAMs is electron-electron interactions between neighbouring molecules, making it more likely that this phenomenon is intrinsic to the Blatter radical.

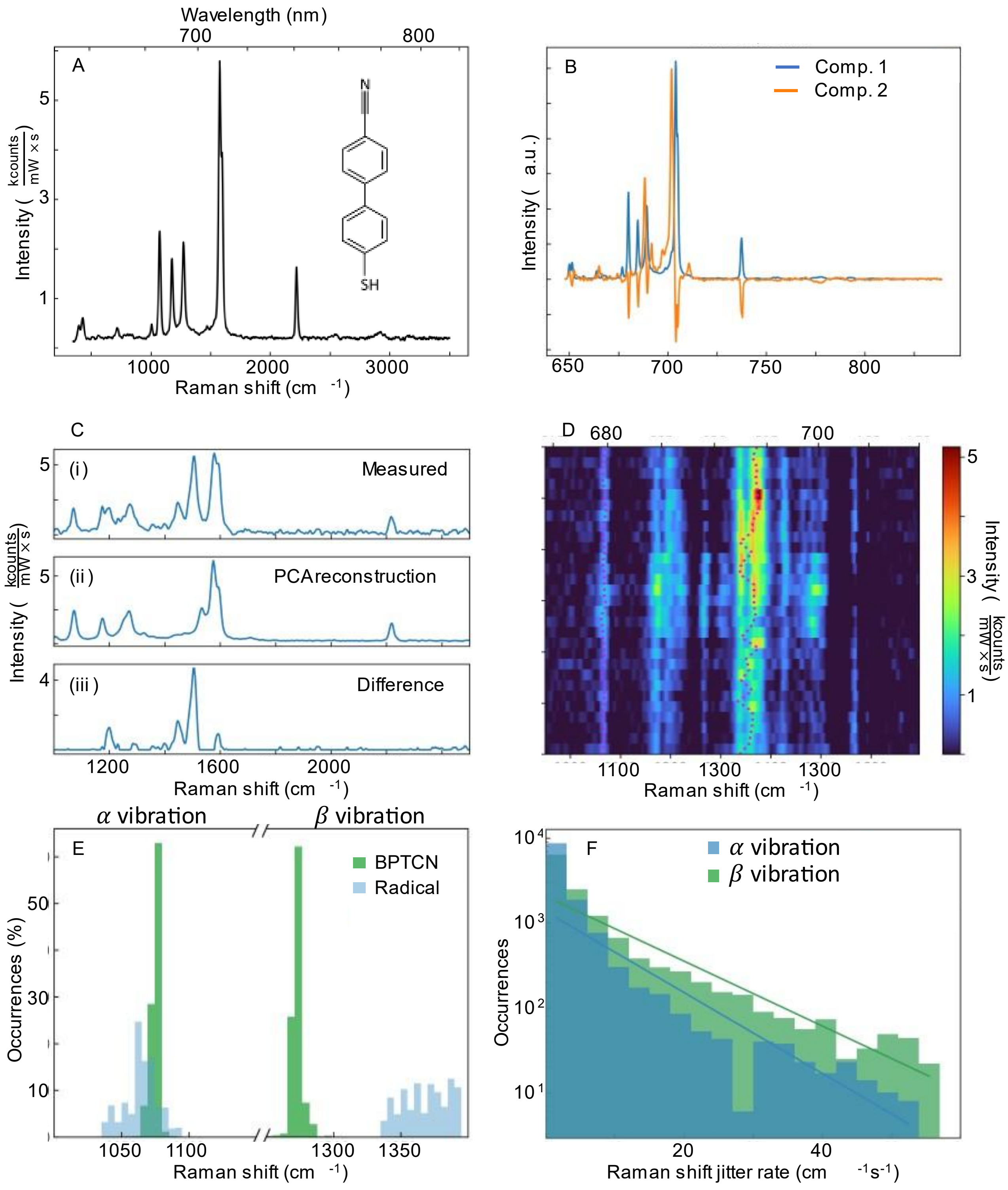

Figure S7. Influence of molecular separation on SERS time-jitter of radical molecule. (A) Average SERS spectrum of BPTCN. Inset: BPTCN molecular structure. (B) individual components of the Principal component analysis (PCA) of BPTCN SERS spectra. (C) Procedure

for extracting radical SERS from mixed SAM data: (i) original data, (ii) PCA reconstruction from components of pure BPTCN sample, (iii) Remaining spectrum after subtracting the reconstructed data. (D) Processed spectrogram of mixed SAM, α and β vibrations indicated by dotted line. (E) Raman shift histograms for a pure BPTCN sample (green) and radical data (blue). Radical produces much broader distribution. (F) Raman shift jitter rate histogram, revealing average jitter rate similar to that of radical SAMs.

Cyclic voltammetry of radical SAMs

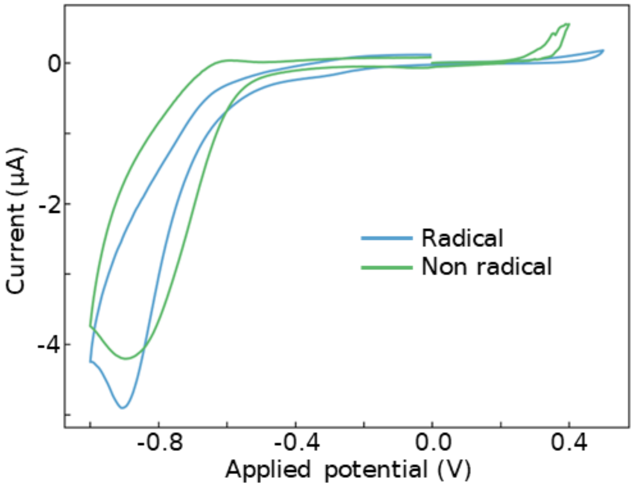

Figure S8. Cyclic voltammetry of Blatter radical (blue) and closed-shell (non-radical) reference molecule (green). Reduction peak seen at ~-0.9 V for both molecules while molecules do not reach full oxidation at +0.5 V. Scan rates are 0.1 V/sec for radical and 0.05 V/sec for closed-shell molecule. 3rd trace shown.

Effect of redox on molecular orbitals and SERS efficiency

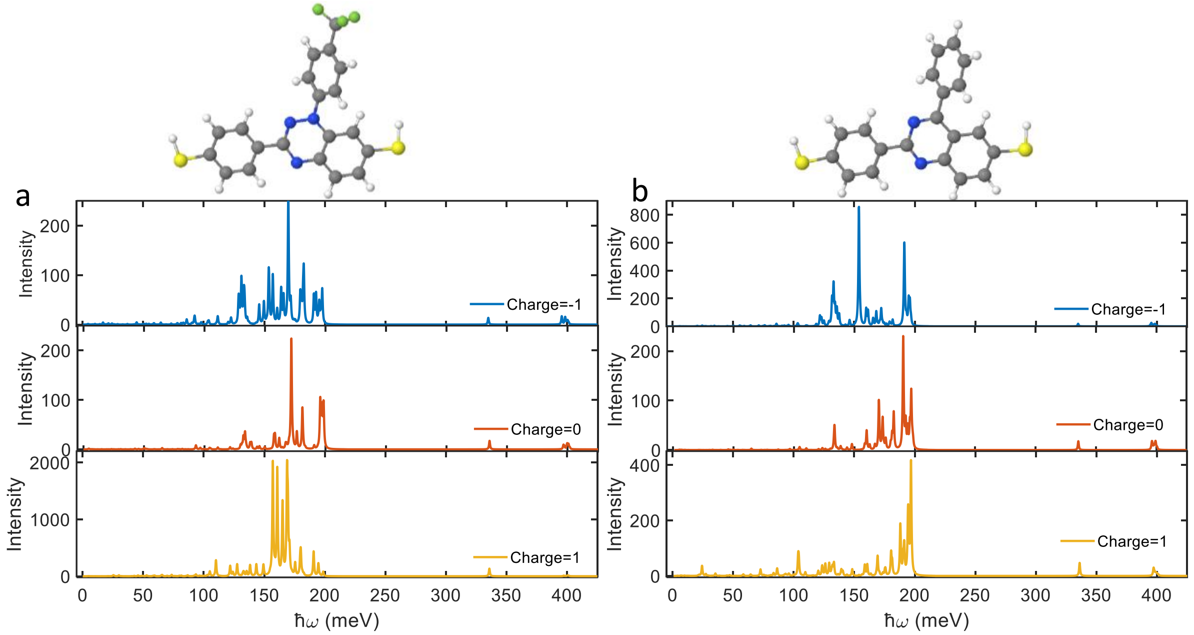

Figure S9. Raman scattering spectrum of (a) Blatter radical and (b) closed-shell (non-radical) compounds in different charge states N (blue N=-1, red N=0, orange N=+1).

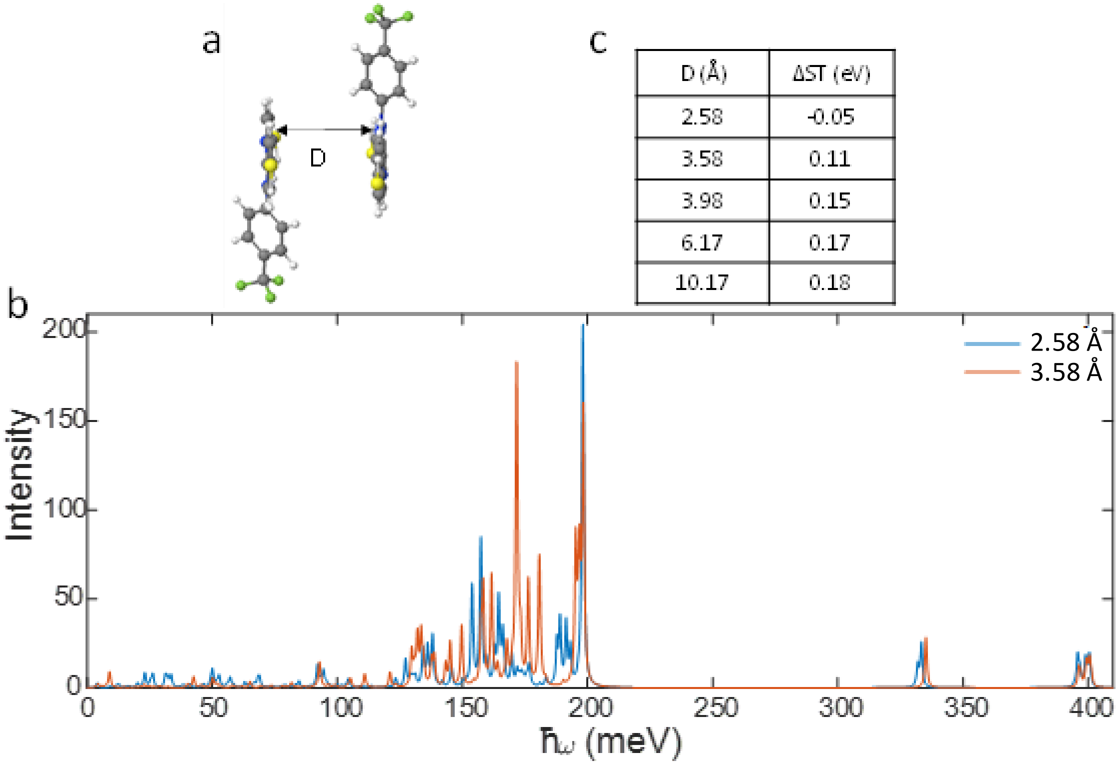

D (Å)	ΔST (eV)
2.58	-0.05
3.58	0.11
3.98	0.15
6.17	0.17
10.17	0.18

Figure S10. Raman scattering spectrum and molecular structure of Blatter radical dimers. (a) Molecular structure of the two radical dimers. (b) Raman scattering spectrum of the dimers in two different distance D from each other. D=3.58 Å is the ground states distance. (c) Table showing ground state energy difference ΔST (eV) between singlet and triplet spin configuration, where ΔST = Total energy of singlet spin state - Total energy of triplet spin state.

References

(1) Xiong, Y.; Huang, J.; Liu, Y.; Xiao, B.; Xu, B.; Zhao, Z.; Tang, B. Z. High-Contrast Luminescence Dependent on Polymorphism and Mechanochromism of AIE-Active (4-(Phenothiazin-10-Yl)Phenyl)(Pyren-1-Yl)methanone. *J Mater Chem C* 2020, *8* (7), 2460–2466. https://doi.org/10.1039/C9TC05064G.

(2) Dar, A. A.; Malik, A. A. Photoluminescent Organic Crystals and Co-Crystals. *J Mater Chem C* 2024, *12* (27), 9888–9913. https://doi.org/10.1039/D4TC01453G.
